# Location, location, location: the evolutionary history of CD1 genes and the NKR-P1/ligand systems

**DOI:** 10.1007/s00251-016-0938-6

**Published:** 2016-07-25

**Authors:** Sally L. Rogers, Jim Kaufman

**Affiliations:** 1 Department of Biosciences, University of Gloucestershire, Cheltenham, GL50 4AZ UK; 2 Department of Pathology, University of Cambridge, Cambridge, CB2 1QP UK; 3 Department of Veterinary Medicine, University of Cambridge, Cambridge, CB3 0ES UK

**Keywords:** 2R, KLRB, CLEC2

## Abstract

CD1 genes encode cell surface molecules that present lipid antigens to various kinds of T lymphocytes of the immune system. The structures of CD1 genes and molecules are like the major histocompatibility complex (MHC) class I system, the loading of antigen and the tissue distribution for CD1 molecules are like those in the class II system, and phylogenetic analyses place CD1 between class I and class II sequences, altogether leading to the notion that CD1 is a third ancient system of antigen presentation molecules. However, thus far, CD1 genes have only been described in mammals, birds and reptiles, leaving major questions as to their origin and evolution. In this review, we recount a little history of the field so far and then consider what has been learned about the structure and functional attributes of CD1 genes and molecules in marsupials, birds and reptiles. We describe the central conundrum of CD1 evolution, the genomic location of CD1 genes in the MHC and/or MHC paralogous regions in different animals, considering the three models of evolutionary history that have been proposed. We describe the natural killer (NK) receptors NKR-P1 and ligands, also found in different genomic locations for different animals. We discuss the consequence of these three models, one of which includes the repudiation of a guiding principle for the last 20 years, that two rounds of genome-wide duplication at the base of the vertebrates provided the extra MHC genes necessary for the emergence of adaptive immune system of jawed vertebrates.

## Introduction and some history

Caesar Milstein, who along with Georges Koehler had discovered and developed monoclonal antibody (mAb) technology, was asked to choose the first antigen for the newly established cluster of differentiation antigen (CD) nomenclature, and he chose CD1 to be the cell surface molecule recognised by the mAb NA1/34 raised against human thymocytes (McMichael et al. 1979; Calabi and Milstein 1986; Martin et al. 1986). After decades of research, an enormous amount about CD1 genes, molecules and functions is now known (reviewed in Porcelli and Modlin 1999; Brigl and Brenner 2004; Salio et al. 2014; Mori et al. 2016, among many others). A most important point for this review is the oft-mentioned fact that the CD1 system has similarities to both the class I and class II systems. In short, the structures of CD1 genes and molecules are much like the class I system, the loading of antigen and the tissue distribution for CD1 molecules are much like the class II system, and phylogenetic analysis places CD1 between class I and class II sequences. This has led noted authorities to suggest that CD1 arose early in evolution, as a third kind of MHC molecule (Martin et al. 1986; Porcelli 1995).

Just as in mammals, the story of CD1 outside of mammals began with a cell surface antigen identified by a mAb. Jim Pickel and Chen-lo Chen in the lab of Max Cooper showed that mAb CB3 raised to chicken bursal lymphocytes recognises on B cells a non-covalent complex of a 44 kDa membrane glycoprotein with chicken β_2_-microglobulin (β_2_m) with the help of mAb from Karsten Skjødt and Jan Salomonsen in the lab of Morten Simonsen (Pickel et al. 1990). At the Basel Institute for Immunology, Jan Salomonsen, convinced that this cell surface molecule would be the avian CD1, used the CB3 mAb to purify the “Pickel protein” from chicken bursal cells, but the work lurched forward with the discovery of a single expressed sequence tag (EST) in one of the first EST libraries. There followed much cloning of cDNA and genomic clones from CB chickens (with MHC haplotype B12), as well as several other inbred experimental lines. It took some years to show that there were two non-polymorphic chicken CD1 genes in tandem and in the same transcriptional orientation, both of which could be expressed as proteins on the cell surface, one of which (CD1.1) indeed is recognised by the mAb CB3 (Salomonsen et al. 2005).

Using genetic mapping to find the CD1 region, Salomonsen and his colleagues were surprised to discover that the two CD1 genes mapped to the B locus, which contains the chicken MHC. At this point, a whole genome shotgun sequence for a red jungle fowl (one of the ancestors of the domestic chicken, *Gallus gallus* domesticus) became available (ICGSC 2004), which showed that the two CD1 genes were only 50 kb from the C4 gene at the end of the previously sequenced BF-BL region (Salomonsen et al. 2005). In the meanwhile, two other groups mined EST libraries and the red jungle fowl (chicken) genome to find CD1 genes that were located at one end of the B21 MHC region (Miller et al. 2005; Maruoka et al. 2005). Sadly, the three papers used slightly different nomenclatures (CD1.1 and CD1.2 in Salomonsen et al. are called chCD1-1 and chCD1-2 in Miller et al., but CD1.2 and CD1.1 in Maruoka et al.); we will use the Salomonsen designations throughout this review. Now, this region has been completely sequenced in one B haplotype (Shiina et al. 2007) and integrated into an overall view of the chicken MHC (Kaufman 2013; Miller and Taylor 2016).

The latest additions to this story include genome mining that yields recognisable CD1 genes from several reptile species (Yang et al. 2015), including one sequence from the lizard green anole (AncaCD1; *Anolis carolinensis*), two expressed sequences from the Chinese alligator (AlsiCD1.1 and AlsiCD1.2, and several others by Southern blot; *Alligator sinensis*) and one expressed sequence and another intact sequence not found to be expressed from the Siamese crocodile (CrsiCD1.1 and CrsiCD1.2, as well as one clear pseudogene and many other sequences by Southern blot; *Crocodylus siamensis*). Similarly, CD1 sequences have been identified in three marsupials, a single gene each in the bandicoot (IsmaCD1; *Isoodon macrourus*) and the Tasmanian devil (SahaCD1; *Sarcophilus harrisii*), but a single pseudogene in the American opossum (ModoCD1; *Monodelphis domestica*) (Baker and Miller 2007; Cheng and Belov 2014). New opportunities for analysis are also provided by the low coverage genomes for many bird species that have recently been published (Jarvis et al. 2014; Zhang et al. 2014).

### CD1 genes are present in mammals, birds and reptiles, but isotypes, binding sites, recycling motifs and genomic locations are not well-conserved

The genes reported in marsupials, chickens and reptiles were identified as CD1 genes based on a suite of characters, although each report differs in exactly what was analysed (Baker and Miller 2007; Cheng and Belov 2014; Salomonsen et al. 2005; Miller et al. 2005; Maruoka et al. 2005; Yang et al. 2015). The analyses included phylogenetic trees after sequence alignment, levels of polymorphism, hydrophobicity of putative binding grooves and presence of recycling motifs in cytoplasmic tails, and tissue distribution and cellular expression. While these genes had the broad characteristics consistent with the CD1 genes of placental mammals (that is to say, eutherians), some features were not conserved in detail or at all.

Phylogenetic trees of either nucleotide or amino acid sequences show that all the CD1 genes from mammals, chickens and reptiles cluster together in a clade separate from the classical class I genes. Many other non-classical class I genes in vertebrates were found not to cluster with CD1 sequences, including the MIC, Mill and MR1 genes of mammals, the YF genes of chickens, the XNC genes of *Xenopus* and related frogs, and various fish genes. Interestingly, two analyses found that CD1 genes are a sister clade for the endothelial protein C receptor (ProCR) genes in humans and chickens (Maruoka et al. 2005; Papenfuss et al. 2015), an interesting finding of uncertain meaning. Anticipating the discussion below, ProCR genes are found on human chromosome 20, chicken chromosome 7 and anole chromosome 3 but not in *Xenopus* or zebra fish; some of the genes around ProCR are the same in chickens and anole but not in humans, and there is no obvious relationship to the MHC paralogous regions. All the CD1 genes in marsupials, chickens and reptiles have intron-exon structures consistent with eutherian mammals. The level of polymorphism has only been assessed for the chicken CD1.2 gene, for which it is extremely low.

The eutherian CD1 genes are found as isotypes that differ in binding pockets and lipids bound, recycling motifs and presence in different intracellular vesicles, cell expression and tissue distribution, receptors on responding cells and function. Within placental mammals, genes of a particular isotype are more closely related between species than they are to other isotypes in the same species; for instance, human CD1D is more like mouse CD1d than like human CD1A, B, C and E. However, none of the CD1 sequences from marsupials, chickens and reptiles are equivalent to any of the five isotypes of placental mammals, as assessed by phylogenetic analysis for the whole nucleotide sequence or for the protein sequence of the whole protein or any of the domains. In essence, the whole nucleotide and protein sequences reported clustered by taxon and separate from placental mammals: the three marsupial isotypes together, the two chicken isotypes (CD1.1 and CD1.2) together and the three reptile isotypes together (with the crocodilian CD1.1 sequences together and CD1.2 sequences together, separate from lizard sequence). However, analysis of the extracellular domains placed chickens separate from reptiles for the α1 domain, but within the reptile cluster for the α2 and α3 domains (Yang et al. 2015). Such relationships are well-known for classical class I sequences, in which a birth and death model of evolution leads to expansion from a particular gene in each group of animals, so that isotypes can be less related between groups of animals than within a particular group (Nei and Rooney 2005; Eirín-López et al. 2012).

The putative binding grooves formed by the α1 and α2 domains of all the CD1 genes were predicted from the sequences to be predominantly hydrophobic, more than those of mammalian classical class I molecules (although not more so than some chicken classical class I molecules, Salomonsen et al. 2005). Such hydrophobic regions are consistent with binding lipids as do eutherian CD1 molecules, and this was tested explicitly by X-ray structures for the extracellular regions of chicken CD1.1 and CD1.2 expressed in insect cells (Zajonc et al. 2008; Dvir et al. 2010). These structures show that both molecules have the same general structure as human CD1 molecules but differ in the details of the binding site. Based on the endogenous ligand from the insect cells as well as modelling and binding of exogenous ligands, the CD1.1 molecule has a large hydrophobic groove that binds lipids with two hydrophobic tails (like human CD1 molecules), with a 16 carbon chain in the F′ pocket and much longer (up to 50 carbons) chain in a long wrap-around A′ pocket that allows the end to extrude into an A′ cleft, along with the potential for substitutions on the main lipid to bind into three cavities along the A′ pocket. In contrast, CD1.2 has a single small A′ pocket (centrally located compared to the two CD1.1 pockets) which is restricted by the two α-helices being closer together as well as by various bulky amino acid side chains at the ends so that CD1.2 can only accommodate up to 16 carbons. This antigen-binding pocket that is so much smaller than those of humans was considered by the authors to be more primordial.

All expressed CD1 sequences have cytoplasmic tails, and many have potential recycling motifs. All human CD1 molecules have very short cytoplasmic tails, with a four amino acid tyrosine-based motif just before the end of the sequence in CD1B, CD1C and CD1D (YQNI, YQDI, YQGV) but not for CD1A and CD1E. The same is true for the expressed marsupial sequences, IsmaCD1 and SahaCD1 which end with putative tyrosine-based motifs (YEGI, YEDM), with the ModoCD1 pseudogene sequence terminated before the transmembrane region. The two crocodilian CD1.1 sequences also have short tails with putative tyrosine-based motifs (both YQDI), but the two CD1.2 sequences are different, with two leucines at the C-terminus of a short cytoplasmic tail (a putative di-leucine motif) in AlsiCD1.2, but a potential tyrosine-based motif (YTRP) in the middle of a longer tail in CrisiCD1.2 (as well as a C-terminal glycine-leucine). The lizard CD1 also has a longer tail, with a potential tyrosine-based motif (YEDV) in the middle. The chicken molecules also differ, with a short tail ending in leucine-isoleucine (a putative di-leucine motif) for CD1.2, but a potential tyrosine-based motif (YGGC) in the middle of a much longer tail in CD1.1 (found in Salomonsen et al. 2005; Miller et al. 2005 but not in Maruoka et al. 2005). It seems likely that the marsupial, lizard and crocodilian CD1.1 cytoplasmic tails contain real tyrosine-based motifs as in human CD1 molecules, but it is not obvious for the other sequences with less canonical motifs located in the middle of longer tails. The consequences in terms of depth of recycling have not been determined for any CD1 molecule outside of mammals, except for chicken CD1.1 which co-localised with fluorescent ovalbumin but not transferrin, likely trafficking to late endosomes or lysosomes like human CD1B and CD1D (Ly et al. 2010).

The expression of CD1 genes in tissues and cells was examined to differing extents in each study, although overall they were not inconsistent with mammals. Endpoint reverse-transcriptase PCR (RT-PCR) showed that RNA was very well expressed in spleen for chicken CD1.1 and CD1.2 (shown also by Northern blot), SahaCD1 (and CrsiCD1.1 and CD1.2 by quantitative RT-PCR), as well as lymph node for SahaCD1, thymus for IsmaCD1, and bursa for chicken CD1.1 and 1.2. Weaker expression was found for SahaCD1 in the lung and uterus, and much weaker for IsmaCD1 in the spleen. Good expression was found for both chicken isotypes in the bone marrow, thymus, lung and the ileum from intestine (and liver for chicken CD1.2), as well as in splenic B cells and CD8 (but not CD4) T cells. Weaker expression of CrsiCD1.1 and CD1.2 was found by quantitative RT-PCR for lung, kidney and small intestine. Expression at the protein level has only been determined for chicken CD1.1 molecules. The mAb CB3 (that recognises chicken CD1.1, Salomonsen et al. 2005) stained all B lymphocytes from bursa, a subpopulation of cells in spleen and blood, and a tiny proportion of cells in thymus, but not T cells (Pickel et al. 1990). A later study showed that the mAb NL1-1.A1 raised to chicken CD1.1 gives similar patterns of staining, with the CD1-bearing cells in the thymus located in class II-high CD3-low cells scattered in the medulla (dendritic cells or medullary epithelial cells) and putative Langerhans cells in the skin, as well as elsewhere (Ly et al. 2010).

Thus, it appears that the CD1 molecules found in marsupials, chickens and reptiles do not correspond exactly to the mammalian CD1 isotypes, but that many of the features are similar, with differences particular to each species presumably reflecting the spectra of pathogens encountered (Dascher and Brenner 2003). The discovery of only a single CD1 molecule in the genomes of the bandicoot, the Tasmanian devil and the green anole suggests that the complex system of isotypes found in mammals is not essential for all vertebrates, and the presence of only a CD1 pseudogene in the opossum genome may mean that the CD1 system is dispensable. Of course, more CD1 genes may exist that have not been found in the genomes thus far, and/or other non-classical class I molecules may have evolved to fill the same purpose. However, unequivocally, one aspect of the CD1 molecules outside of mammals gave a big surprise: the genomic location.

## The genomic location of CD1 genes varies in different species

The human CD1 genes are found together in (or near, see argument below) an MHC paralogous region on chromosome 1 (also known as the CD1 region), with the CD1 genes in other mammals in comparable (syntenic) regions. In addition to the human MHC on chromosome 6, there are (at least) three other MHC paralogous regions that have many genes (so-called paralogues) similar to those in the MHC, located on chromosomes 1, 9 and 19. The existence of these four paralogous regions was taken by Masanori Kasahara (Kasahara et al. 1997) (and independently by another group, Katsanis et al. 1996) as support for the 2R hypothesis, originally proposed by Susumo Ohno (Ohno 1970) and later given support by the group of Peter Holland (Garcia-Fernandez and Holland 1994), that there had been two rounds of genome-wide duplication near the base of the vertebrates, beginning some 600 million years (My) ago. Moreover, Kasahara proposed that class I genes were present in the primordial MHC before duplication so that class I genes would initially be distributed to the MHC paralogous regions, and at some later point in time, CD1 genes might arise by duplication in one of the paralogous regions, followed by differential loss so that eventually classical class I genes would be located in the MHC and CD1 genes in the CD1 region (and perhaps another non-classical class I gene, the Fc receptor of neonates, FcRn, in a third paralogous region, Kandil et al. 1996).

**Fig. 1:**
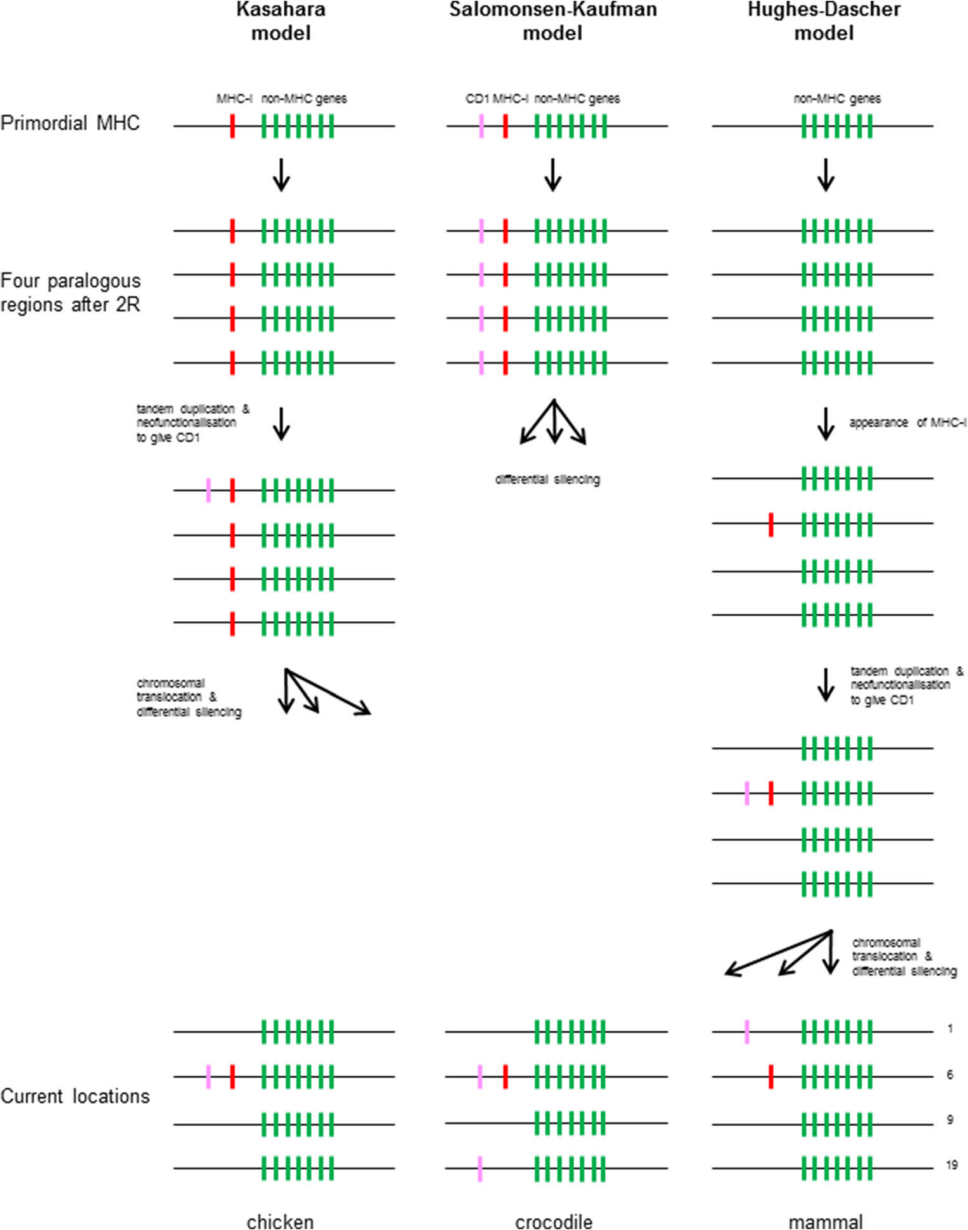
Three models have been proposed for the origin and subsequent evolution of CD1 genes. Lefthand column, the Kasahara model, in which classical MHC class I genes emerge in a primordial MHC before the two rounds of genome-wide duplication, then a duplication in one paralogous region gives rise to the gene that evolves into a CD1 gene, followed by differential silencing to give classical class I genes in the MHC and CD1 genes in the CD1 region of mammals, but requiring chromosomal translocations to put CD1 onto other chromosomes in birds and reptiles. Middle column, the Salomonsen-Kaufman model, in which classical MHC class I genes emerge in a primordial MHC and then one class I gene evolves into a CD1 gene, followed by two rounds of genome-wide duplication, with differential silencing to give both classical class I genes and CD1 genes in the MHC of the lineage that led to birds, classical class I genes in the MHC and CD1 genes in the CD1 region in the lineage leading to mammals, and classical class I genes in the MHC but CD1 in various regions in reptiles. Righthand column, the Hughes-Dascher model, in which the two rounds of genome-wide duplication are followed by the emergence of classical MHC class I genes in a primordial MHC next to one paralogous region, with a duplication in the MHC giving rise to the gene that evolves into a CD1 gene, with this arrangement persisting the lineage leading to birds, but with various translocations of CD1 genes out of the MHC near to a paralogous regions in the lineages leading to reptiles and mammals. Genes considered to part of the ancient paralogous region except for class I genes (*green bars*), classical class I genes (MHC-I) (*red bars*), and CD1 genes (*pink bars*). Paralogous regions are in order of the chromosome number found in humans: *1*, chromosome 1; *6*, chromosome 6 (MHC); *9*, chromosome 9; *19*, chromosome 19 (colour figure online)

The biggest surprise from the discovery of chicken CD1 genes was their genomic location at the edge of the BF-BL region, the chicken MHC, rather than in the MHC paralogous region equivalent to chromosome 1 in humans. Three models have been suggested to explain the location of CD1 genes (Fig. 1), the first being the model by Kasahara and colleagues just mentioned (Kasahara et al. 1997).

In a second model (Fig. 1), Salomonsen, Kaufman and colleagues (Salomonsen et al. 2005) interpreted this finding to mean that the CD1 genes had likely arisen by duplication from the classical class I genes already in the primordial MHC before duplication and that the differential silencing had occurred independently in the lineage leading to mammals and the lineage leading to birds, so that CD1 was present in the CD1 region of mammals but in the MHC of birds. If this scenario is true, then CD1 genes might have been expected in one or more of any of the MHC paralogous regions in other lineages. In fact, this is what has been found for the reptiles; in particular the CD1 genes are present in both the MHC and a region identified as a chromosome 19-paralogous region in alligators and crocodiles (Yang et al. 2015). However, this model cannot explain why no CD1 genes have been identified in the amphibian *Xenopus* or in the fish species examined.

In a third model, Miller, Dascher and colleagues cited the phylogenetic trees reported by Hughes as well as their own unpublished phylogenetic trees to argue that CD1 molecules arose in a common ancestor of birds and mammals, around 310 My ago, although they did mention the Kasahara model as an alternative (Hughes 1991; Miller et al. 2005). The phylogenetic trees that formed the basis for this view can give a topology of evolution, but the timescale is dependent on the rate of substitution, which is notoriously difficult to determine in genes that are under strong selection for a new function. However, the eventual model (Fig. 1) proposed by Chris Dascher (Dascher 2007) was that the MHC with classical MHC genes only arose after the two rounds of genome-wide duplication at the base of the vertebrates, that a CD1 gene arose in this single MHC by duplication from classical class I genes followed by neofunctionalisation to bind lipids and that the CD1 gene(s) were then translocated to another chromosome during the fragmentation and reassembly of chromosomes early in the lineage leading to mammals. Thus, the presence of human CD1 genes near the MHC paralogous region on chromosome 1 was ascribed to chance, with the point being made that this paralogous region was broken into two halves anyway. In this model, the presence of the CD1 genes in the MHC is the ancestral situation, which would be found in reptiles (and perhaps in amphibians or even the ancestors of terrestrial tetrapod vertebrates).

What evidence might allow us to decide between these three alternative models for the appearance of CD1 genes? There are three general points to be made.

First, the Hughes-Dascher model predicts (and the Kasahara model can be consistent with) the lack of CD1 genes in most or all fish. In fact, no CD1 sequence has been found in any amphibian or fish by a variety of analyses. Among the most sensitive has been a search based on a hidden Markov model (HMM) that found all of the known class I genes (including CD1 genes in mammals and chicken) and predicted many other class I genes that have been verified but failed to find any identifiable CD1 genes in the genomes of the lizard green anole, the frog *Xenopus tropicalis* and fish including zebra fish *Danio rerio*, pufferfish *Tetraodon* and lamprey *Petromyzon marinus* (Papenfuss et al. 2015).

However, there are at least four other explanations for the lack of CD1 genes identified in amphibians and fish. A trivial explanation is that the genomes are incomplete, as highlighted by the failure of the HMM to identify a CD1 gene in the green anole (Papenfuss et al. 2015), which was later found in a more complete genome. Correct long-range assembly is another issue, with a recent example being the fact that the organisation and chromosomal locations of so-called B30.2 genes only began to make sense with the most recent release of the zebra fish genome sequence (Howe et al. 2016). A third possibility is that CD1 did exist in fish but has been lost in favour of another nonclassical class I molecule which can perform the same function; in this regard, the chicken YF molecule appears to bind lipids much like CD1 (Afanassieff et al. 2001; Hee et al. 2010). A fourth explanation might be that CD1 genes arose early in evolution but simply have been lost in extant amphibians and fish. A precedent for such a loss in intervening animals is the antibody heavy chain isotype IgD, which was long thought to be specific to the mammalian lineage, until IgD genes were discovered first in fish (along with the related IgW genes), then in the frog *Xenopus*, and eventually in reptiles (Ohta and Flajnik 2006; Gambón-Deza and Espinel 2008; Fillatreau et al. 2013). Discovery of a CD1 gene in some fish could be accommodated by both the Kasahara model and the Salomonsen-Kaufman model.

Second, the Hughes-Dascher model also predicts that the primordial MHC appeared after (not before!) the two rounds of genome-wide duplication at the base of the vertebrates. This prediction is consistent with the fact that no classical MHC genes, T cell receptor (TcR) genes, antibody genes or recombination activating genes (RAGs) have been discovered in jawless fish (Uinuk-Ool et al. 2002) (although genes that could be ancestors of TAP, TcR and CD4 have been described in lampreys, Uinuk-Ool et al. 2003; Pancer et al. 2004). However, in lampreys and hagfish, an analogous adaptive immune system based on genes encoding leucine-rich repeats (LRRs), the variable lymphocyte receptors (VLRs) have been described which are expressed by cells similar to B cells and T cells, the latter spending time in parts of the gills which express some genes like the vertebrate thymus, so-called thymoids (Pancer and Cooper 2006; Boehm et al. 2012). Again, it could be argued that the genes for the adaptive immune system were present in the common ancestor of the jawless fish and jawed vertebrates, and were then lost in the jawless fish, but that argument would require these genes to be lost from all MHC paralogous regions.

In contrast, both the Kasahara model and the Salomonsen-Kaufman model predict that the classical MHC genes arose in a proto-MHC before the two rounds of genome-wide duplication. Indeed, the Salomonsen-Kaufman model is part of a larger model that was based on their discovery of an NK cell receptor gene called B-NK in the chicken MHC (Kaufman et al. 1999), which they used to suggest that not only the classical MHC genes as ligands but also their antigen processing/ peptide loading genes and their receptors were all present in primordial MHC, which would be the birthplace of the adaptive immune system (Rogers et al. 2005; Walker et al. 2011, Kaufman 2011). This view of the primordial MHC was echoed by Flajnik and Kasahara in their epic review (Flajnik and Kasahara 2010), citing the discovery of genes very similar to TAP transporter and tapasin genes in an MHC syntenic region of amphioxus, a key protochordate. However, no sequences recognisable as class I genes were found in amphioxus by the sensitive HMM method, as described above (Papenfuss et al. 2015).

Third, the Hughes-Dascher model predicts that the CD1 gene(s) landed near an existing paralogous region during the fragmentation and rearrangement of chromosomes in the lineage leading to mammals, and therefore are not really a part of the MHC paralogous region on human chromosome 1 (nor would the FcRn be part of the paralogous region on human chromosome 19). It is not quite clear when this movement might have taken place, since the CD1 pseudogene on chromosome 2 in opossum is clearly located in a region with genes syntenic to the human CD1 region, while the CD1 gene of Tasmanian devils is reported to be on the same chromosome as the MHC (Baker and Miller 2007; Cheng and Belov 2014). The question of whether the FcRn gene is in or near an MHC paralogous region is even less clear. From ENSEMBL, the single FcRn gene (FCGRT) is found on human chromosome 19, opossum chromosome 4 and on a scaffold in Tasmanian devil, all very close to the genes NOSIP, RCN3 and RPS11. No such gene has been found by searching the chicken genome, but chickens do have a function for translocation of IgY to the egg. However, it depends on a molecule similar to phospholipase A2 receptor (West et al. 2004), encoded by a gene (PLA2R1) located on chromosome 7 in the chicken genome; of course this could be an example of another molecule assuming the function of a non-classical class I molecule (as mentioned above for YF and CD1).

**Fig. 2:**
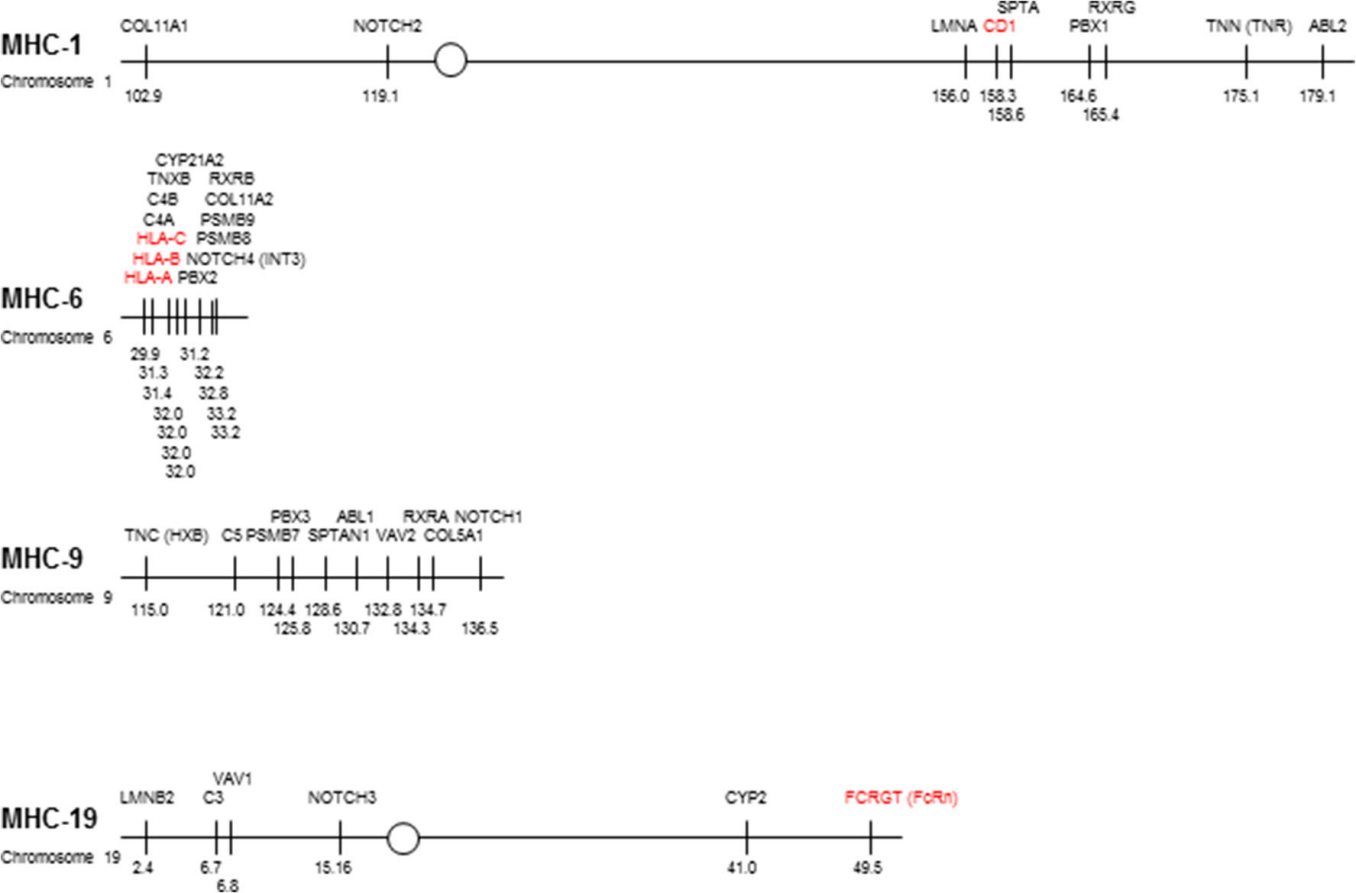
The human MHC paralogous regions differ significantly in size, with the CD1 genes in the middle of a large region on chromosome 1 relative close to paralogous genes, the classical class I genes on the edge of a small region on chromosome 6 very close to paralogous genes, and the FcRn gene relatively far away from one end of paralogous genes on chromosome 19. Comparison of the four paralogous regions based on the genes originally proposed by Kasahara (along with the FcRn or FCGRT gene, but with those deemed later to be questionable excluded), roughly to scale with the chromosomal position according to the current assembly of the human genome (GRCh38.p5). *Circles* indicate centromeres, *thin lines* indicate gene(s) considered to be paralogous (many tens to thousands of other genes not shown), and positions are given in megabases (Mb) according to EMSEMBL (www.ensembl.org/Homo_sapiens). Class I genes (*red*) (colour figure online)

The increasingly broad understanding of potential paralogous regions coupled with constant refinement of the human genome sequence might help to determine whether the CD1 and FcRn genes were originally present in the region that was twice duplicated to become paralogous regions, or were translocated nearby after the appearance of the paralogous regions. The simplest (and most extreme) examples might be that there is a cluster of tightly linked paralogous genes with either the CD1/FcRn genes embedded in the middle and obviously part of the paralogous region, or located some way off and obviously not part of the paralogous region. Sadly, the situation is more complex than is usually presented (Fig. 2). The genes that were originally identified as being paralogous (Kasahara 1997) are located within a 3.2-Mb region in the MHC on human chromosome 6 but are spread over roughly 21 to 76 Mb in each of the paralogous regions, with these paralogous genes separated by many tens of other genes that are not obviously paralogous (as found by locating these genes in ENSEMBL). More recent analyses, taking into account other organisms (Kasahara 2000; Abi-Rached et al. 2002; Azumi et al. 2003; Danchin and Pontarotti 2004; Kasahara et al. 2004; Suurväli et al. 2014), improve but do not substantially change this view (with interesting details outside the scope of this review). The selective identification of genes considered to be paralogous in a sea of genes that are not obviously paralogous (particularly for genes that are part of a large multigene family with members throughout the genome) certainly led to skepticism for the early claims for two rounds of genome-wide duplication at the base of the vertebrates (for instance, Hughes 1998), which was largely dispelled once the amphioxus genome became available (Putnam et al. 2008).

The CD1 genes are found in the middle of the 76 Mb covering the original paralogous genes on chromosome 1, relatively close to some well-defined paralogous genes such as LMNA, SPTA1, PBX1 and RXR. This finding is most easily accommodated by the CD1 genes being part of the region before duplication, but it remains possible that the CD1 genes were inserted into the middle of an existing paralogous region. In contrast, the FcRn gene is located at one end of the 47-Mb region containing the paralogous genes on chromosome 19, relatively far away (and across the centromere) from the bulk of those paralogous genes (particularly as determined by later analyses, for instance Suurväli et al. 2014). Thus, examination of the human genome does not rule out any of the proposed models but it would seem most parsimonious for CD1 genes to be present before the genome-wide duplications and consistent with FcRn gene appearing later in evolution.

Although it might be plausible that the CD1 genes moved from the MHC to a different chromosome in the lineages leading to mammals, another potential challenge to be explained by the Hughes-Dascher model is the presence of reptile CD1 genes in more than one chromosomal location (Yang et al. 2015), specifically in both the MHC (like some birds) and near to genes identified as similar to the paralogous region on human chromosome 19. One possibility is that the assemblies of these genomes are not accurate, and further work will show that all these genes are together in the MHC. Another possibility is that there have been multiple translocations out of the MHC, with the reptile CD1 genes landing near an MHC paralogous region on the equivalent of human chromosome 1 in mammals as well as near an MHC paralogous region on the equivalent of human chromosome 19. A third possibility is that the Kasahara and Salomonsen-Kaufman models are more parsimonious explanations for the data.

**Fig. 3:**
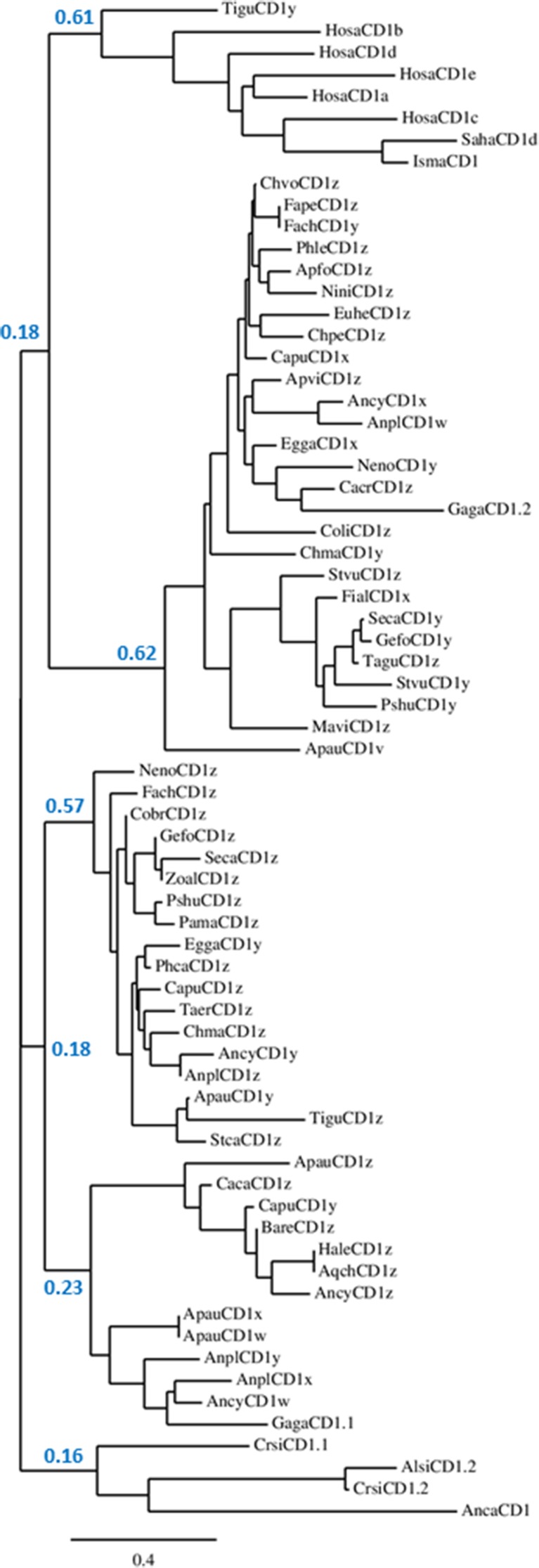
Neighbour-joining tree showing relationships for a selection of CD1 genes from eutherian and marsupial mammals, birds and reptiles. Alignment of full length amino acid sequences was done using MUSCLE. Confidence for major nodes indicated; other tree-building algorithms gave the same major clades of mammals (but including one tinamou sequence), reptiles and two avian clades, but joined with different topologies. Species indicated as follows: Alsi, *Alligator sinensis* (Chinese alligator); Anca, *Anolis carolinensis* (Carolina anole lizard); Ancy, *Anser cygnoides domesticus* (swan goose); Anpl, *Anas platyrhynchos* (mallard duck); Apau, *Apteryx australis mantelli* (North island brown kiwi); Apfo, *Aptenodytes forsteri* (emperor penguin); Apvi, *Apaloderma vittatum* (bar-tailed trogon); Aqch, *Aquila chrysaetos Canadensis* (golden eagle); Bare, *Balearica regulorum gibbericeps* (grey crowned crane); Caca, *Caprimulgus carolinensis* (chuck-will’s widow); Cacr, *Cariama cristata* (red-legged seriema); Capu, *Calidris pugnax* (ruff); Chma, *Chlamydotis macqueenii* (MacQueen’s bustard); Chpe, *Chaetura pelagica* (chimney swift); Chvo, *Charadrius vociferous* (killdeer); Cobr, *Corvus brachyrhynchos* (American crow); Coli, *Columba livia* (rock dove); Crsi, *Crocodylus siamensis* (Siamese crocodile); Egga, *Egretta garzetta* (little egret); Euhe, *Eurypyga helias* (sun bittern); Fach, *Falco cherrug* (Saker falcon); Fape, *Falco peregrinus* (peregrine falcon); Fial, *Ficedula albicollis* (collared flycatcher); Gaga, *Gallus gallus* (chicken); Gefo, *Geospiza fortis* (medium ground finch); Hale, *Haliaeetus leucocephalus* (bald eagle); Hosa, *Homo sapiens* (human); Isma, *Isoodon macrourus* (northern brown bandicoot); Mavi, *Manacus vitellinus* (green collared manakin); Neno, *Nestor notabilis* (kea); Nini, *Nipponia Nippon* (Japanese crested ibis); Pama, *Parus major* (great tit); Phca, *Phalacrocorax carbo* (great cormorant); Phle, *Phaethon lepturus* (white-tailed tropicbird); *Picoides pubescens* (downy woodpecker); Pshu, *Pseudopodoces humilis* (ground tit); Saha, *Sarcophilus harrisii* (Tasmanian devil); Seca, *Serinus canaria* (Atlantic canary); Stca, *Struthio camelus australis* (ostrich); Stvu, *Sturnus vulgaris* (common starling); Taer, *Tauraco erythrolophus* (red-crested turaco); Tagu, *Taeniopygia guttata* (zebra finch); Tigu, *Tinamus guttatus* (white-throated tinamou); Zoal, *Zonotrichia albicollis* (white-throated sparrow)

Recently, low-coverage whole genome sequences have been reported for a wide variety of birds (Jarvis et al. 2014; Zhang et al. 2014). It must be kept in mind that many important genes will be absent in such partial genomes and that there will be many assembly errors for those genes that are present. However, cursory analysis finds CD1 genes in all bird genomes available (nearly 100 genes from over 40 species), ranging from the Palaeognathae (ostrich, kiwi and tinamou) to the Passeriformes (songbirds) of the Neoaves. Phylogenetic analysis (Fig. 3) of all available CD1 sequences (full length protein) gives four clades: one for mammals (both eutherian and marsupial, but surprisingly with one CD1 sequence from tinamou), one for reptiles, and two for birds, one with chicken CD1.1 and one with chicken CD1.2 (with most birds having at least one of each), suggesting that both avian isotypes were present in an early ancestor.

**Fig. 4:**
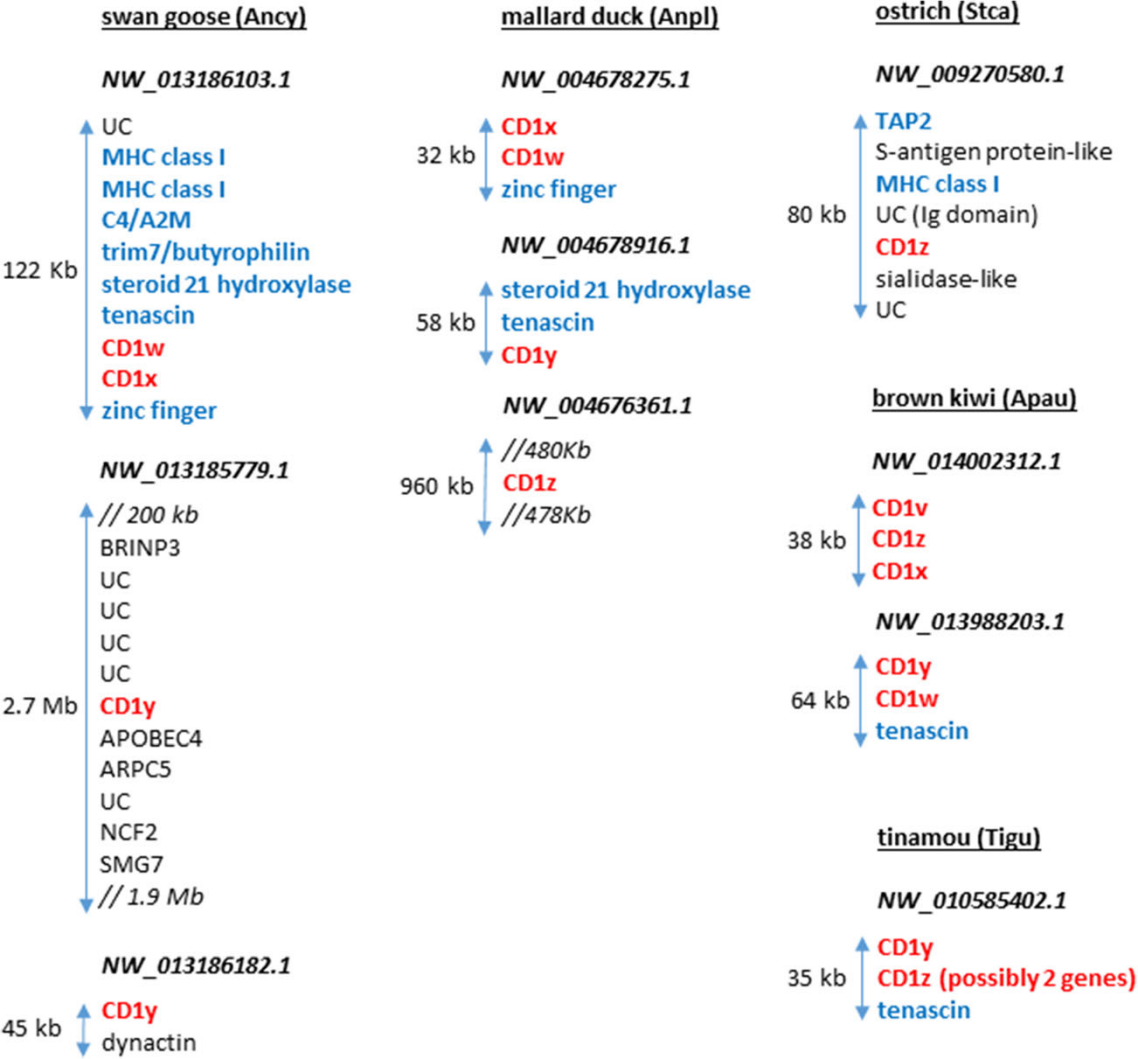
The relative position of CD1 genes on scaffolds from selected avian genomes is shown in comparison to other genes. Genes were identified (S. Rogers, unpublished) using the ENSEMBL genome browser (www.ensembl.org), and most likely orthology of the gene confirmed by blasting back against the NCBI database (www.ncbi.nlm.nih.gov). All potential CD1 genes (*red bold*) are indicated, designated with letters going backwards from z. In addition, MHC (*blue bold*) genes as well as other genes (*black*) are shown, with uncharacterised predicted proteins labelled UC. The tinamou CD1 gene that groups with mammals in phylogenetic analyses is labelled CD1y, and the other CD1 gene on that contig may in fact be two genes, one of which is more closely related to an MHC class I gene. Lengths of contigs are shown with *arrows*, and where other genes are inserted is indicated by double forward slash (//), with the distance shown if significant. *kb* kilobases, *Mb* megabases (colour figure online)

Examination of the existing genome sequences reveals that avian CD1 genes are typically found on one to three contigs, each with one to three CD1 genes. To our surprise, only in the most primitive of bird species (Palaeognathae which include the flightless ratites, and Galloanseres of the Neoaves which include chickens and ducks) are the CD1 genes found next to recognisable MHC genes (Fig. 4). For instance, white throated tinamou (*Tinamus guttatus*), considered to be most primitive of all birds (Jarvis et al. 2014), has two putative CD1 genes next to each other with tenascin, with one of the CD1 genes midway in sequence between CD1 and classical class I genes. The ostrich (*Struthio camelus australis*) has a single CD1 gene together with TAP2 and class I genes, and the north island brown kiwi (*Apteryx australis mantelli*) has five CD1 genes, three together without other recognisable genes, and two together with tenascin. The mallard duck (*Anas platyrhynchos*) has four CD1 genes on three contigs, two together with a zinc finger, one with tenascin and one with nothing recognisable. The swan goose (*Anser cygnoides domesticus*) also has four CD1 genes on three contigs, two together with tenascin, TRIM7/butyrophilin-like, C4/α_2_-macroglobulin-like and two MHC class I genes, one with APOBEC4, and one with dynactin. This last constellation of genes is most intriguing, with class I genes typical of the mammalian class I region, tenascin typical of the mammalian class III region, TRIM7 and butyrophilin typical of the mammalian extended class I region, and α_2_-macroglobulin found in mammalian natural killer complex (NKC). As mentioned above, it has been suggested long ago that the NKC and the MHC were originally adjacent, as part of the primordial MHC (Rogers et al. 2005). For the bird lineage, it would appear that the ancestor had CD1 genes in the MHC (as well as other genomic locations), which were then lost from the MHC in the Neoaves.

Overall, it would appear that the CD1 genes can be found in the MHC and more than one of the MHC paralogous regions, apparently even in the same species. One explanation would be that CD1 genes were present in all four paralogous regions in the last common ancestor of reptiles, birds and mammals, and have been differentially silenced in each lineage, indeed in each species. However, if CD1 genes were present in all four paralogous regions, were they originally present in amphibians and the many thousands of different fish species, and are now lost? Another explanation might be that CD1 genes can move around in the genome, perhaps preferentially between genomic regions with some level of sequence homology, as might be expected for MHC paralogous regions. This possibility leads us to consider another unexpected finding in the chicken MHC, the presence of the lectin-like NK receptor and potential ligand.

## The genomic location of lectin-like NK receptor and ligand genes vary in different species

One of the many surprises that arose from the first genomic sequence of the chicken MHC was the presence of two genes that encoded lectin-like domains with apparent transmembrane exons located as in type II membrane proteins, located head-to-head in opposite transcriptional orientation. The one called B-NK was very clearly most closely related to a well-defined NK receptor in mammals, NKR-P1, and was found to be expressed in chicken NK cell clones (Kaufman et al. 1999). The other called B-lec was found to be closely-related to early activation antigens such as CD69, but most closely to human LLT1 and mouse clr genes, which were eventually shown to be ligands of NKR-P1 (Rogers et al. 2005). Subsequently, a sequencing facility renamed the genes, so in their view, B-lec became Blec1, B-NK became Blec2, and further genes with high identity to B-lec became Blec3, Blec4 and so on (Shiina et al. 2007). This nomenclature sadly removes both the relative relationships and the functional associations that are so critical to any deep understanding of the biology. A more appropriate nomenclature has been established in which NKR-P1 genes are part of the KLRB1 family, and genes related to LLT1/clr/B-lec are part of the CLEC2 family (Carlyle et al. 2008; Kirkham and Carlyle 2014), a nomenclature which we will adopt when appropriate for the rest of this review.

In mammals, NKR-P1 (KLRB1) gene(s) and ligand (CLEC2) gene(s) are found to be located (typically in receptor-ligand pairs) in the natural killer complex (NKC), together with other lectin-like receptor genes such as KLRG1 (also known as MAFA) and other signature genes such as TMEM52b, GABARAPL1 and α_2_-macroglobulin. The NKC is located on chromosome 12 in humans, so not with the immunoglobulin-like NK receptors in the leukocyte receptor complex (LRC) on chromosome 19 or in the MHC on chromosome 6, or near to any of the MHC paralogous regions (Carlyle et al. 2008; Kirkham and Carlyle 2014). In fact, two chicken lectin-like genes are found in a large genomic region along with the genes for α_2_-macroglobulin, TMEM52b and GABARAPL1, but neither lectin-like gene is an NK receptor (Chiang et al. 2007; Neulen and Göbel 2012).

As for the CD1 genes, we proposed that B-NK and B-lec were present in the primordial MHC before the two rounds of genome-wide duplication, and then these genes were differentially silenced in different paralogous regions of the lineage leading to mammals and to birds. We proposed that the NKC and the MHC were originally one region, in which both receptors and ligands were present in order to co-evolve (Rogers et al. 2005; 2008; Kaufman 2011); this idea has been picked up by others (Flajnik and Kasahara 2010).

We found that the B-NK gene had high allelic polymorphism (with a different allele for each MHC haplotype), while B-lec was virtually monomorphic (Rogers and Kaufman 2008). Unexpectedly, no evidence was found that B-NK recognised B-lec (Viertlboeck et al. 2008), but multiple genes with high sequence identity to B-lec were found in the TRIM and BG regions next to the MHC and in the unlinked Rfp-Y region on the same chromosome (Rogers et al. 2003; Shiina et al. 2007; Salomonsen et al. 2014), any of which could be ligands for B-NK. Sequences for quail also showed a variety of B-lec genes (Shiina et al. 2004).

**Fig. 5:**
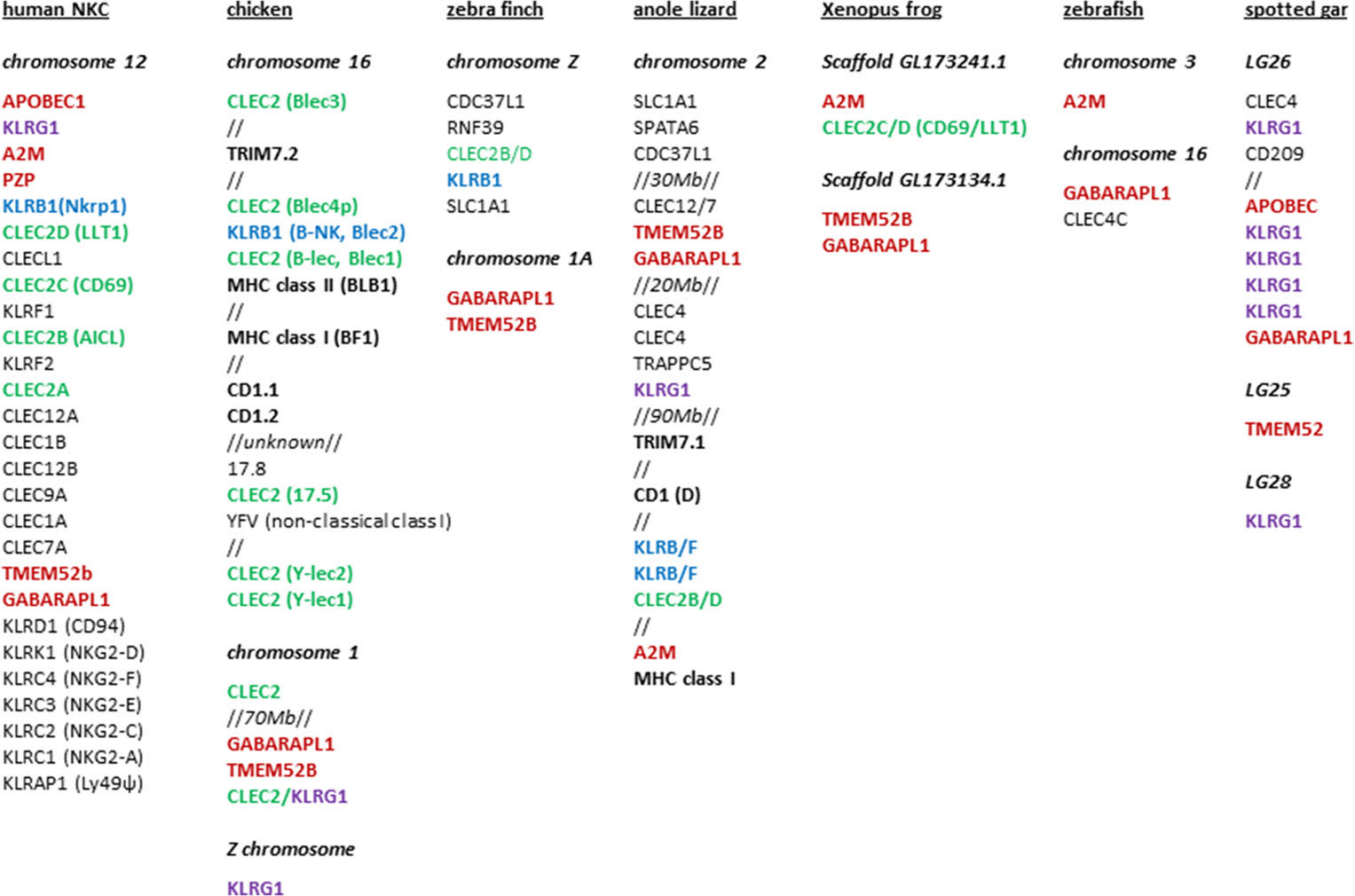
The relative positions of CLEC2 and KLRB genes on chromosomes (or linkage groups, LG) in different species are shown in comparison to other genes. Genes were identified (S. Rogers, unpublished) using the ENSEMBL genome browser (www.ensembl.org), and most likely orthology of the gene confirmed by blasting back against the NCBI database (www.ncbi.nlm.nih.gov). In some species, the marker gene could not be identified or located and therefore it is not shown. All potential CLEC2 (*green bold*) and KLRB (*blue bold*) genes, as well as selected KLRG (*purple bold*), NKC (*red bold*) and MHC (*black bold*) genes identified in the various species are shown. Where other genes are inserted is indicated by double forward slash (//), with the distance shown if significant (*Mb* megabases) (colour figure online)

It seemed possible to explain the appearance of a multigene family of B-lec genes with a single B-NK gene in and around the MHC of chickens and other galliforms, but it was a shock to find that the B-NK and B-lec gene pair in a passerine bird, the zebra finch (*Taeniopygia guttata*), is located on the Z sex chromosome (Ekblom et al. 2011). A further analysis using the most current zebra finch genome (taeGut3.2.4) reveals a CLEC2 gene and an unannotated KLRB1 gene next to SLC1A1 and CDC37 on the Z chromosome (Fig. 5), with no lectin-like genes near the TMEM52b and GABARAPL1 genes in chromosome 1, a region otherwise syntenic with the NKC. Similarly, the genome (Ellegren et al. 2012, FicAlb_1.4 version 84.1) of another passerine bird, the collared flycatcher (*Ficedula albicollis*), has both a KLRB1 gene and a CLEC2 gene in between SLC1a1 and CDC37L1.

Despite the fragmentary nature of the data (as discussed above), the low-coverage whole genome sequences for a wide variety of birds (Jarvis et al. 2014; Zhang et al. 2014) highlight the possibilities for a variety of locations for KLRB1 and CLEC2 genes. In the emperor penguin, for example, KLRB1-CLEC2 gene pairs are associated with a TRIM7 gene (which in mammals is located in the extended MHC) and with TMEM52b and GABARAPL1 (which in mammals are found in the NKC). Another eight CLEC genes are found in this putative NKC, as well as a KLRG1 gene next to α_2_-macroglobulin on a separate scaffold. Overall, KLRB1-CLEC2 gene pairs or singletons are found associated with genes that in mammals are found in the NKC, the extended MHC and/or the Z chromosome. Interestingly, the Z chromosome is believed to share a common ancestor with human chromosome 9 (Bellott et al. 2010), which also contains an MHC paralogous region. Thus, one possibility is that a piece of an MHC paralogous region became part of the Z chromosome in birds and was silenced in some bird lineages but not in passerine birds.

In the genome sequence of the lizard, the green anole (Alföldi et al. 2011, AnoCar2.0 version 84.2), one KLRG1 gene, two KLRB1 genes and one CLEC2 gene are found on chromosome 2, along with many genes found in the mammalian NKC (including TMEM52b, GABARAPL1 and α_2_-macroglobulin), the MHC of chickens and/or mammals (including two class I genes, a CD1 gene and TRIM7.1) and the avian Z chromosome (including SLC1A1). Although this collection of genes is spread over 150 Mb, their presence all on the same chromosome is consistent with the idea that all three regions in birds were once together in a reptilian ancestor.

The presence of KLRB1-CLEC2 gene pairs is less clear in amphibians and fish, although there is some evidence for an NKC. In the genome sequence of the frog, *Xenopus tropicalis* (Hellsten et al. 2010, JGI 4.2 version 84.42), a single CLEC2 gene is located next to α_2_-macroglobulin, with TMEM52b and GABARAPL1 located on another scaffold, suggestive of either incomplete assembly or a fragmented NKC. In a representative of the teleost fish, the zebra fish, the genome sequence (Howe et al. 2013, GRCz10 version 84.10) had no obvious KRLB1 or CLEC2 genes, and of the genes characteristic of the NKC, GABARAPL1 and α_2_-macroglobulin were found but on separate chromosomes. The genome sequence of a less derived fish, the spotted gar (Braasch et al. 2016, LepOcu1 version 84.1), also had no obvious KRLB1 or CLEC2 genes, but there were four lectin-like receptor genes very similar to KRLG1 in between GABARAPL1 and APOBEC genes, along with another KLRG1 gene close by, suggesting that an NKC was already present in basal fish (and possibly lost in teleost fish). Sadly, examination of the elephant shark genome (Venkatesh et al. 2014, accession number AAVX02000000) gave no indication of KLRG1, KRLB1 or CLEC2 genes, or of a syntenic region for the NKC (although this genome is particularly poorly assembled).

Overall, the evidence is most consistent with the idea that the lectin-like genes of the NKC were present very early, likely in the primordial MHC before two rounds of genome-wide duplication, and that these genes were differentially silenced in different paralogous regions in different taxa. The alternative is that these genes move around selectively between particular regions of the genome. There is an enormous literature about recombination, translocation, chromosomal breakpoints and so on during evolution (as just one example in chickens, Romanov et al. 2014), far beyond the scope of this review. The important point to note is that these genes, ones that seem to be part of ancient MHC paralogous regions but in fact might not be, would have moved selectively near or into paralogous regions. Whether regional sequence identity or the presence of particular sequence features might contribute to such selective movement is again beyond the scope of this review. However, it is also possible that the appearance of such genes near ancient MHC paralogous regions is only due to our expectations and that when rigorous statistical analyses are carried out, the location of these genes will turn out to be random.

## The way backwards

Our review of the evolution of CD1 and certain lectin-like NK cell receptors leaves more questions than answers. Even for the few marsupial, bird and reptile CD1 genes known, there is a great deal to learn through comparative immunology. In particular, the cells in which each of these molecules are expressed, the recycling patterns that the molecules undergo, the nature of their binding sites and the kinds of lipids that actually bind these molecules, and the cells that recognise the lipid-CD1 complexes all remain to be discovered. Some approaches should be relatively straightforward. Expression of CD1 molecules will allow structural studies (like those already done for chicken CD1) as well as the generation of monoclonal antibodies which can be used to determine tissue expression, study cell biology and isolate molecules to determine the lipids bound, which in turn will allow the production of lipid-CD1 multimers to discover the responding cells. A crucial but much more difficult step is to determine the role of each of these CD1 molecules in response to particular diseases (or for normal regulation of the immune response, if that is important). Once all of these tasks are accomplished, it may become possible to pick out the important patterns that have governed the details of CD1 evolution, in order to understand which features have been selected and which features are due to chance. The journey to understand the lectin-like NK receptors and their ligands is likely to be similar but much more complicated, given the enormous complexity of NK cell biology.

Overshadowing the comparative biology of both the CD1 and lectin-like NK receptors is the question of their genomic histories. For nearly 20 years, a guiding principle for the evolution of adaptive immunity has been the concept of genome-wide duplications at the base of the vertebrates leading to the extra genes that provided the potential to evolve an adaptive immune system. A large literature has explored this concept (although there are as many reviews as actual data papers), and it remains a powerful way to organise and explain the disparate data that have been gathered. However, the Hughes-Dascher model completely repudiates this view, with the original adaptive immune system of jawed vertebrates envisaged to have appeared next to one of the paralogous regions after the two rounds of genome-wide duplication. Is it possible, after all of the sound and fury, that genome-wide duplication is in fact irrelevant to the origin and evolution of the adaptive immune system of the jawed vertebrates?

Despite our own published models and beliefs, the existing data do not allow us to choose definitively between the dogma of Kasahara and the catastrophic antithesis of Hughes and Dasher. The analogous adaptive immune system of VLRs discovered in jawless fish does not provide clear evidence one way or the other, since the jawless fish have undergone at least one round of genome-wide duplication (Flajnik and Kasahara 2010). However, two other analogous systems have been reported: fibrinogen-related proteins (FREPs) in molluscs which gain somatic diversity by point mutation (Zhang et al. 2004; 2008) and Down syndrome cell adhesion molecule (DSCAMs) in arthropods which gain somatic diversity through alternative splicing (Schmucker and Chen 2009). If FREPs and DSCAMs are confirmed as adaptive immune systems among invertebrates (Ng et al. 2014; Armitage et al. 2015; Gordy et al. 2015), then genome-wide duplications are not necessary for the emergence of adaptive immunity. However, such duplications may still have played an important role in the emergence of our own adaptive immune system. Only time (and more research) will tell.
